# The predatory mite *Typhlodromalus aripo* prefers green-mite induced plant odours from pubescent cassava varieties

**DOI:** 10.1007/s10493-012-9595-0

**Published:** 2012-06-29

**Authors:** Alexis Onzo, Rachid Hanna, Maurice W. Sabelis

**Affiliations:** 1 Biological Control Centre for Africa, International Institute of Tropical Agriculture, 08 B.P. 0932, Cotonou, Benin, West Africa; 2 Institute for Biodiversity and Ecosystem Dynamics, University of Amsterdam, Kruislaan 320, 1098 SM Amsterdam, The Netherlands; 3 Faculté d’Agronomie, Université de Parakou, B.P. 123, Parakou, Benin; 4 IITA-Benin, c/o IITA Ltd., 26 Dingwall Road, Croydon, CR9 3EE UK

**Keywords:** Herbivore-induced plant volatiles, Y-tube olfactometer, Phytoseiidae, Olfactory preference, Tetranychidae, Africa

## Abstract

It is well known that plant-inhabiting predators use herbivore-induced plant volatiles to locate herbivores being their prey. Much less known, however, is the phenomenon that genotypes of the same host plant species vary in the attractiveness of these induced chemical signals, whereas they also differ in characteristics that affect the predator’s foraging success, such as leaf pubescence. In a series of two-choice experiments (using a Y-tube olfactometer) we determined the preference of *Typhlodromalus aripo* for pubescent versus glabrous cassava cultivars infested with the cassava green mite *Mononychellus tanajoa* and also the preference for cultivars within each of the two groups. We found that when offered a choice between pubescent and glabrous cassava cultivars (either apex or leaves), *T. aripo* was significantly more attracted to pubescent cultivars. For each cultivar, *M. tanajoa* infested leaves and apices were equally attractive to *T. aripo*. There was however some variation in the response of *T. aripo* to *M. tanajoa*-infested plant parts within the group of pubescent cultivars, as well as within the group of glabrous cultivars. Our study confirms not only that *T. aripo* uses herbivore-induced plant volatiles to search for prey in cassava fields, but it also shows that it can discriminate between glabrous and pubescent cultivars and prefers the latter. This knowledge can be useful in selecting cultivars that are attractive and suitable to *T. aripo*, which, in turn, may promote biological control of the cassava green mite.

## Introduction

It is now widely accepted that plants can promote the effectiveness of the natural enemies of their herbivores (Price et al. 1980; Sabelis et al. 1999, 2007; Cortesero et al. 2000). They protect themselves against phytophagous arthropods in many ways, ranging from chemical (e.g. toxins, digestibility reducers), to morphological (e.g. pubescence, tissue toughness, refuge) defences that either limit herbivore attacks or increase the efficiency of the natural enemies of herbivores (Dicke and Sabelis 1988; Barret 1994; Walter 1996; Sabelis et al. 1999; Norton et al. 2001). Plants can attract the natural enemies of herbivores by emitting specific volatile chemicals when attacked by herbivores. These volatile chemicals, generally referred to as herbivore-induced plant volatiles (HIPV), are among the main information-conveying agents available to predatory arthropods when searching for prey (Dicke et al. 1990a; Turlings et al. 1990; Sabelis et al. 2007; Onzo et al. 2009).

The quantity and quality of HIPV are affected by many factors such as leaf age, herbivore species, time of the day, light intensity, season and water stress, plant species and cultivar (Takabayashi et al. 1994; Turlings et al. 1995). Several studies have shown that plant genotypes or cultivars of a given plant species produce different blends of HIPV (Turlings et al. 1995; Krips et al. 2001; Scutareanu et al. 2001, 2003). Loughrin et al. (1995) reported that the amount and the composition of emitted volatiles were qualitatively different between commercial and naturalized cotton infested by the beet armyworm, *Spodoptera exigua* (Hübner). Takabayashi et al. (1991) reported that blends emitted by apple leaves infested with spider mites from either of two different species *Tetranychus urticae* Koch and *Panonychus ulmi* (Koch) differed mainly in quantitative composition for some compounds. In contrast, they observed that the blends emitted by leaves of two apple cultivars infested by the same spider mite species—*T. urticae*—differed significantly both quantitatively and qualitatively (see also Shiojiri et al. 2010 for a similar case).

Sabelis and van de Baan (1983) and Dicke et al. (1990b) observed that the response of *Phytoseiulus persimilis* Athias-Henriot to the volatiles emitted by two bean cultivars (*Phaseolus vulgaris* L.) infested by the two-spotted spider mite *T. urticae* showed a significant difference in their attractiveness to the predatory mite *P. persimilis*. More recent studies on pear cultivars (Scutareanu et al. 2001, 2003), on gerbera cultivars (Krips et al. 2001) and on maize cultivars (Gouinguené et al. 2001; Tamiru et al. 2011), indicated that different cultivars of the same plant species produce different volatile blends when infested by the same herbivore species. Volatile blends can also differ depending on herbivore genotype even when attacking the same cultivar of plant (Kant et al. 2008).

Field observations in Africa showed that the frequency and abundance of the predatory mite, *Typhlodromalus aripo* De Leon, differed among cassava cultivars infested by the cassava green mite (CGM) *Mononychellus tanajoa* (Bondar). This predatory mite inhabits the apex (growing point) of cassava plant branches during day-time, but during night-time it commutes between apex and leaves to forage for CGMs, its prey (Onzo et al. 2003). Cassava apex traits such as size, compactness, and pubescence—particularly the latter—matter to the abundance of *T. aripo*, this predator is more frequently found and more abundant on cassava cultivars with pubescent apices compared with cultivars with glabrous apices (Hanna et al. 2000; Zundel et al. 2009). As one of the possible explanations for the differential abundance of *T. aripo* on the various cultivars, we hypothesize that pubescent cultivars produce HIPV that is more attractive to the predatory mite *T. aripo* than glabrous cultivars. In this study, we use olfactometry to test whether adult females of *T. aripo* prefer odours of CGM-infested pubescent cultivars over CGM-infested glabrous cultivars.

## Materials and Methods

We used a Y-tube olfactometer (Sabelis and van de Baan 1983; Takabayashi and Dicke 1992) to determine the extent to which *T. aripo* predators are attracted to pubescent and glabrous cassava genotypes. The Y-tube olfactometer consisted of a Y-shaped glass tube with a Y-shaped iron wire positioned in the middle, and parallel to the tube walls to provide a ‘railroad’ to the mites. The base of the Y-tube was connected to an air pump that produced a unidirectional airflow from the arms to the base of the tube. The arms were connected via a plastic tube to two identical plastic boxes each of which contained a test odour sources (e.g. Aratchige et al. 2004).

Preparation of plant parts for use in the Y-tube olfactometer two-choice tests followed the same protocol for all three experiments. Cassava apices and leaves of all seven cultivars used in this study were collected from mite-free cassava plants grown in large pots under semi-field conditions in a screenhouse (Onzo et al. 2005) of the International Institute of Tropical Agriculture in Cotonou, Benin. The potted cassava plants were 4–5 weeks old by the time their leaves and apices were picked for use in the experiments.

Field-collected individuals of *M. tanajoa* were maintained indoors or in a screenhouse on potted cassava plants. Specimens of *T. aripo* used in the experiments were also collected from cassava fields at the IITA-Station or close to IITA campus, then put in paper bags and kept in a refrigerator at c.a. 12 °C in a laboratory before their use in the experiments.

Leaves and apices used as odor sources were detached from cassava plants in the morning and then each with their petiole placed immediately in a water-filled glass vial (0.8 cm in diameter and 4 cm deep), or each with their stem end (10 cm below the apex) in a larger water-filled plastic vial (2.7 cm in diameter and 6.5 cm deep). The vials were sealed with parafilm to keep the plant tissue fresh during the experiment (Gnanvossou et al. 2003). Leaves were collected from the top of the canopy (i.e. leaf 3–4), because green mites prefer young cassava leaves. In the afternoon of the same day, 25 adult females *M. tanajoa* were used to infest each leaf, whereas 5 adult females *M. tanajoa* were used to infest each apex (Gnanvossou et al. 2001; 2003). The *M. tanajoa* infested plant tissues were then incubated in plastic cages (70 cm long, 40 cm wide and 40 cm high), for 3 days under continuous light provided by two fluorescent light sources placed above the cages. Cassava plant parts from different cultivars were incubated in separate cages (but in the same room). For each set of olfactometer experiments, odor source consisted of four *M. tanajoa*-infested leaves or 16 *M. tanajoa*-infested apices to obtain matching amounts of plant biomass (Gnanvossou et al. 2003).

Adult females of *T. aripo* were kept individually in a plastic vial (10 mm diam. and 40 mm long) without food for 2 h prior to the olfactometer bioassays (see Gnanvossou et al. 2001). To investigate the olfactory response of *T. aripo* to the different cassava cultivars, three separate series of Y-tube experiments were conducted. In the first series of experiments we compared the response of *T. aripo* to volatiles from pubescent and glabrous cassava cultivars infested by *M. tanajoa* to determine whether *T. aripo* has a preference. The pubescent genotypes were represented by the cultivars Agric, TMS 92/0326, Oko-Iyawo, whereas the glabrous cultivars were represented by cultivars Gbeze, Odongbo, TMS 30572 and Amala. All these cultivars, except Gbeze, were used in the field studies described by Hanna et al. (2000). Each cultivar in a group (i.e. pubescent or glabrous) was tested against each cultivar in the other group.

In a second series of Y-tube experiments with the same cultivars as in the first, we tested whether the different pubescent (or glabrous) cultivars were equally attractive to *T. aripo*. All combinations of cultivars within each of the two categories (pubescent/glabrous) were compared. Thus, the three pubescent cultivars were compared in two-choice tests as follows: (1) apex of Oko-Iyawo versus apex of TMS 92/0326; (2) apex of Oko-Iyawo versus apex of Agric; and (3) apex of Agric versus apex of TMS 92/0326. Similarly the four glabrous cultivars were compared in two-choice tests as follows: (1) Apex of Gbeze versus apex of TMS 30572; (2) apex of Gbeze versus apex of Odongbo; (3) apex of Gbeze versus apex of Amala; (4) apex of TMS 30572 versus apex of Odongbo; (5) apex of TMS 30572 versus apex of Amala; and (6) apex of Amala versus apex of Odongbo.

In a third series of Y-tube experiments, we compared the attraction of *T. aripo* to leaves and apices of pubescent and glabrous cassava cultivars. The objective was to determine whether attraction of *T. aripo* is to the apex alone or to cassava leaves alone or to both. In this experiment, apices and leaves of the glabrous cultivar Gbeze or those of the pubescent cultivar Agric were used in two-choice tests in the Y-tube olfactometer as follows: (1) apex of Gbeze versus apex of Agric; (2) leaf of Gbeze versus leaf of Agric; (3) leaf of Gbeze versus apex of Agric; (4) apex of Gbeze versus leaf of Agric; and (5) apex of Agric versus leaf of Agric. Because the cultivar Gbeze was relatively less colonized by *T. aripo*, we decided not to test the combination of apex of Gbeze versus leaf of Gbeze.

Each of the two arms of the Y-tube olfactometer had an air stream coming from one of the two boxes, each with a different odor source. Each individual predator was placed at the base of the iron wire positioned in the middle of the Y-shaped glass tube and parallel to the tube walls (Sabelis and van de Baan 1983). The predator was observed until it reached the end of one of the two arms, or for a maximum of 5 min, after which it was removed. After testing a series of five consecutive females, the odor sources were interchanged to correct for any unforeseen asymmetry in the experimental set-up (Sabelis and van de Baan 1983). The number of females reaching each odor source, as well as those that did not make a choice was recorded. In each replicate experiment 20 *T. aripo* females were tested sequentially in two-choice tests in the Y-tube olfactometer and each experiment was replicated three times, taking place on consecutive days. New odor sources were used each day.

### Statistical analysis

To analyze the olfactometer results we used a replicated G-test against the null hypothesis that predators reach the end of the two arms with equal probability (1:1) (Sokal and Rohlf 1981). This test yields three G-statistics: G_P_, G_H_ and G_T_. If G_T_ is significant, then this may due to heterogeneity among replicates. This is why it is important to test for heterogeneity. If G_H_ is not significant, then the conclusions can be based on a G-test on pooled data (G_P_). If the G_H_-test is significant, however, the significance of G_T_ may be due to heterogeneity among replicates. In that case the variation among replicates has to be scrutinized before drawing conclusions (see Aratchige et al. 2004 for an example). Females that failed to walk to the far end of one of the arms were not included in the data subject to the statistical analysis. To facilitate interpretation of the data presented in the Figures, critical P values from binomial tests against a 1:1 hypothesis are provided for the pooled data for each treatment (Zar 1984). Also here, females that failed to walk to the far end of one of the arms were not included in the statistical analysis.

## Results

### Attraction of *Typhlodromalus aripo* to volatiles emitted by apices or leaves of pubescent versus glabrous *Mononychellus tanajoa*-infested cassava cultivars

**Fig. 1:**
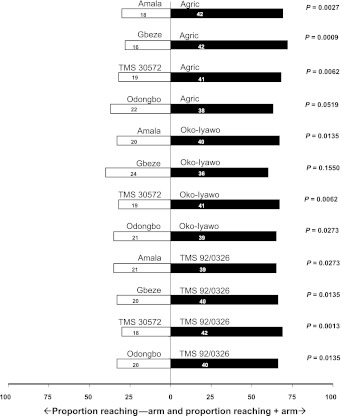
Response of *Typhlodromalus aripo* when offered a choice between volatiles from *Mononychellus tanajoa*-infested apices of pubescent versus glabrous cassava cultivars, in a Y-tube olfactometer. *Numbers* in the *bars* represent the total number of predators that chose either olfactometer arm. To the *right* of each *horizontal bar* critical *P* values are given from a binomial test against a 1:1 null hypothesis applied to the pooled data of three replicate experiments [*Open bars* = % attracted to glabrous apices (−arm); *filled bars* = % attracted to pubescent apices (+arm)]

In all comparisons, *T. aripo* was attracted mainly to apices of the pubescent cultivars (Table 1; Fig. 1). The proportions of predators that chose apices of the pubescent cultivars were statistically larger than those that chose apices of the glabrous cultivars, with two exceptions: the proportions of *T. aripo* females that were attracted to the pubescent cultivar Oko-Iyawo compared with the glabrous cultivar Gbeze and those attracted to the pubescent cultivar Agric compared with the glabrous cultivar Odongbo were, in both sets of choices, numerically larger but not significantly different from a 1:1 ratio (Fig. 1). The replicated G test revealed that heterogeneity among replicate experiments (each with 20 predators) was not significant. Hence, it is justified to base the conclusions on a G-test on pooled data and this showed a significant deviation from a 1:1 ratio, confirming that *T. aripo* predators prefer the apices of pubescent cultivars over those from glabrous cultivars.

**Table 1:** Results of olfactometer tests for the responses of *Typhlodromalus aripo* to apices of pubescent (+) and glabrous (−) cassava cultivars

Odour sources	No. replicates	n (+)	n (−)	n (0)	N
A. Pubescent apices versus glabrous apices	36	480	238	2	718
B. Pubescent leaves versus glabrous leaves	3	39	19	2	58
C. Pubescent apices versus pubescent apices	9	81	99	0	180
D. Glabrous apices versus glabrous apices	18	166	194	0	360
E. Pubescent apices versus pubescent leaves	3	26	34	0	60
F. Pubescent apices versus glabrous leaves	3	42	17	1	59
G. Pubescent leaves versus glabrous apices	3	39	19	2	58

Number of female predators tested in each replicate experiment is equal to 20

Similar to the tests with cassava apices, *T. aripo* showed a significant preference for infested leaves from the pubescent cultivar Agric to infested leaves from the glabrous cultivar Gbeze (Table 1; Fig. 4). Since heterogeneity among replicate experiments was not significant, the G-test on pooled results was done and this showed a significant deviation from a 1:1 ratio confirming that the *T. aripo* predators prefer odour of leaves from the pubescent cultivar over odour from leaves a glabrous cultivar (Table 2).

**Table 2:** Results of the replicated goodness-of-fit tests (G-test) for the responses of *Typhlodromalus aripo* to apices of pubescent (+) and glabrous (−) cassava cultivars

Odour sources	Source								
	Pooled			Heterogeneity			Total		
	df	G-statistics	Critical level	df	G-statistics	Critical level	df	G-statistics	Critical level
A. Pubescent apices versus glabrous apices	1	83.18	*P* < 0.001	35	11.15	*P* > 0.25	36	94.33	*P* < 0.001
B. Pubescent leaves versus glabrous leaves	1	7.04	0.005 < *P* < 0.01	2	0.44	*P* > 0.25	3	7.48	0.05 < *P* < 0.25
C. Pubescent apices versus pubescent apices	1	1.80	0.10 < *P* < 0.25	8	5.73	*P* > 0.25	9	7.53	*P* < 0.25
D. Glabrous apices versus glabrous apices	1	2.18	0.10 < *P* < 0.25	17	23.47	0.10 < *P* < 0.25	18	25.65	0.10 < *P* < 0.25
E. Pubescent apices versus pubescent leaves	1	1.07	*P* > 0.25	2	1.76	*P* > 0.25	3	2.83	*P* < 0.25
F. Pubescent apices versus glabrous leaves	1	10.94	*P* < 0.001	2	0.23	*P* > 0.25	3	11.17	0.10 < *P* < 0.25
G. Pubescent leaves versus glabrous apices	1	7.04	0.005 < *P* < 0.01	2	0.07	*P* > 0.25	3	7.11	0.05 < *P* < 0.25

### Attraction of *Typhlodromalus aripo* to volatiles emitted by apices of pubescent versus pubescent, or glabrous versus glabrous *Mononychellus tanajoa*-infested cassava cultivars

**Fig. 2:**
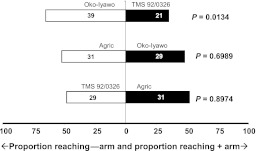
Response of *Typhlodromalus aripo* when offered choice between volatiles from *Mononychellus tanajoa*-infested apices from two pubescent cassava cultivars, in a Y-tube olfactometer. *Numbers* in the *bars* represent the total number of predators that chose either olfactometer arm. To the *right* of each *horizontal bar* critical *P* values are given as obtained by a binomial test against a 1:1 null hypothesis [*Open bars* = % attracted to pubescent apices (−arm); *filled bars* = % attracted to pubescent apices (+arm)]

Among pubescent cultivars, *T. aripo* did not display significant preferences in the comparisons between the cultivars Agric and Oko-Iyawo or between the cultivars Agric and TMS 92/0326. When given a choice between Oko-Iyawo and TMS 92/0326, however, *T. aripo* significantly preferred the former (Table 1; Fig. 2). Since there was no significant heterogeneity among replicate experiments, a G-test on pooled results was carried out and this did not show a significant deviation from a 1:1 ratio (*P* > 0.05), suggesting that the tested predators had no preference (Table 2).

**Fig. 3:**
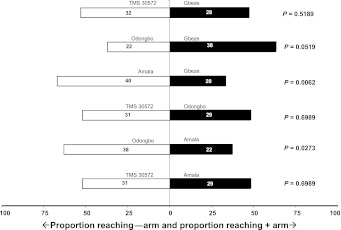
Response of *Typhlodromalus aripo* when offered a choice between volatiles from *Mononychellus tanajoa*-infested apices from two glabrous cassava cultivars, in a Y-tube olfactometer. *Numbers* in the *bars* represent the total number of predators that chose either olfactometer arm. To the *right* of each *horizontal bar* critical *P* values are given from a binomial test against a 1:1 null hypothesis applied to the pooled data of three replicate experiments [*Open bars* = % attracted to glabrous apices (−arm); *filled bars* = % attracted to glabrous apices (+arm)]

Similarly among glabrous cultivars (Table 1; Fig. 3), *T. aripo* did not show a preference in two-cultivar comparisons, except for Odongbo when Amala was the alternative and for Amala when Gbeze was the alternative. The non-preference of *T. aripo* among the glabrous cassava cultivars was also confirmed by the pooled results of the replicated goodness of fit tests (*P* > 0.05; Table 2).

### Attraction of *Typhlodromalus aripo* to apex versus leaf of *Mononychellus tanajoa*-infested cassava plants

**Fig. 4:**
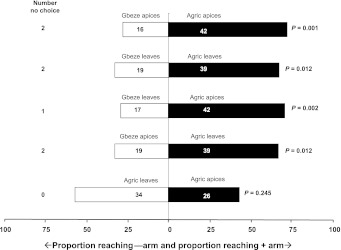
Response of *Typhlodromalus aripo* when offered a choice between volatiles from *Mononychellus tanajoa*-infested apices or leaves of pubescent or glabrous cassava cultivars, in a Y-tube olfactometer. *Numbers* in the *bars* represent the total number of predators that chose either olfactometer arm. To the *right* of each *horizontal bar* critical *P* values are given from a binomial test against a 1:1 null hypothesis applied to the pooled data of three replicate experiments [*Open bars* = % attracted to glabrous apices or leaves (−arm); *filled bars* = % attracted to pubescent apices or leaves (+arm)]

The predator showed no significant preference when offered infested leaves versus infested apices of the pubescent cultivar Agric (Table 1; Fig. 4). When offered the choice between infested leaves of the glabrous cultivar Gbeze and those of the pubescent cultivar Agric apices, or between Gbeze apices and Agric leaves, *T. aripo* were significantly more attracted to Agric (whether apex or leaf) (Table 1). These results were also confirmed by the replicated G-tests (Table 2). Since there was no significant heterogeneity among replicate experiments, we proceeded to do a G-test on pooled data and these showed that the predators had a significant preference for odour from infested parts of pubescent cultivars (Table 2).

## Discussion

Our study shows that—in 10 out of 12 two-choice comparisons between pubescent and glabrous cassava cultivars (Fig. 1)—*T. aripo* preferred pubescent cultivars and that the predator uses HIPV to locate its preferred host plant. This general preference of *T. aripo* for pubescent rather than glabrous cassava cultivars provides at least a partial explanation for the greater abundance of *T. aripo* in the field on pubescent rather than glabrous cultivars (Hanna et al. 2000). Colonization of pubescent cassava cultivars by *T. aripo* is not, therefore, by chance. Pubescence may increase the relative humidity inside the apex, thereby improving the survival conditions for its residents, especially during the dry season (R. Hanna, personal communication). Whereas it is known from previous studies that residing in the apex provides to *T. aripo* protection against UV-induced mortality (Onzo et al. 2010); it is not yet demonstrated, however, that pubescence reinforces this protection. A novel finding emerging from this study is that the predator uses the volatiles emitted from leaves as well as those from apices. This response may enhance the predator’s ability to find its preferred host plant since it is expected that the quantity of volatile blends emitted by leaves is far greater than those by apices alone, because the leaf biomass of a cassava plant is larger than that of its apices.

The differential response of *T. aripo* to pubescent and glabrous cassava cultivars suggests a difference in the composition and/or in the amount of the volatile cues emitted by each of the cassava cultivars, as observed by Hoballah et al. (2002) and Tamiru et al. (2011) for plant varieties with completely different system of host-plant species (maize), phytophagous insect (caterpillar) and natural enemy (parasitoid insect). Pubescent cultivars appeared to produce volatile blends that are more attractive to the predatory mite *T. aripo* than blends produced by glabrous cassava cultivars. Our study also showed some differences in within-group comparisons, notably the clear preference for Oko-Iyawo when tested against TMS 92/0326 in comparisons including only pubescent cultivars, and the clear preference for Odongbo over Amala and Amala over Gbeze in comparisons including only glabrous cultivars. On the basis of findings from comparisons within the glabrous group it was expected that *T. aripo* would have clear preference for Odongbo when tested against Gbeze, since Odongbo was more attractive than Amala, and Amala was more attractive than Gbeze. The results were more close to the opposite, however: there was a numerically higher attraction (and a nearly significant response; *P* = 0.0519) to Gbeze compared with Odongbo. This may be due to differences in the relative composition or in the amount of volatiles produced by these three glabrous cultivars. Further studies are needed to compare the chemical composition of HIPV emitted by different cassava cultivars under attack by *M. tanajoa* to determine which compounds or which blend of compounds (see van Wijk et al. 2011) are responsible for the attraction of *T. aripo.* Variation in quantity and quality of odor may well explain within- cultivar group variability in *T. aripo* attraction to pubescent and glabrous cassava cultivars.

### Implications for the cassava production system in Africa

Morphological plant characteristics may play an important role in the biological control of agricultural crop pests (Skirvin and Fenlon 2001). They may not only affect herbivores but also their natural enemies (Sabelis and Bakker 1992; Barret 1994; Dicke 1995). Our findings suggest that cassava plant breeders should not only consider the effect of pubescence on the second trophic level but also that on the third trophic level. Thus, by using knowledge of plant-natural enemy interactions instead of aiming only at developing pest/disease-resistant cultivars, plant breeders may produce cultivars with a higher yield under realistic field conditions. In doing so, they may contribute to enhancing predator efficiency by breeding plants emitting a more effective blend of HIPV in that it attracts more natural enemies or helps arresting them on the plants. In selecting a cultivar, plant breeders should, therefore, aim at improving conditions that increase predator-searching behavior thereby increasing the effectiveness of plant pest control (Dicke et al. 1990b). Plant volatiles, as well as plant morphological characteristics, may play an essential role in plant–herbivore–predator interactions and are therefore also promising targets for improved crop protection (Kant et al. 2009).
